# Surgical treatment of non-unions in the ulna and radius: a one-year outcome study

**DOI:** 10.1007/s00068-025-03024-0

**Published:** 2025-11-25

**Authors:** Sebastian Findeisen, Katinka Maier, Thomas Ferbert, Michael Tanner, Michael Tanner, Tobias Grossner, Tim Niklas Bewersdorf, Christian Schamberger, Gerhard Schmidmaier, Jessica Carina Böpple

**Affiliations:** Clinic for Trauma and Reconstructive Surgery, University Hospital Heidelberg, Schlierbacher Landstraße 200a, Heidelberg, 69118 Germany

**Keywords:** Non-union, Forearm non-union, Bone regeneration, Non-union treatment, Autologous bone

## Abstract

The incidence of non-unions of the forearm following conservative or surgical treatment of forearm fractures ranges from 2% to 5.3%. These non-unions can lead to significant limitations in the patient’s physical capabilities, necessitating an appropriate treatment plan. The aim of this study was to evaluate osseous consolidation of forearm non-unions after six months and one year as well as to assess patient-reported outcomes. A total of 36 patients with non-unions of the forearm, operatively treated in our department between 01/10 and 12/21, completed the follow-up period for this study. Radiographic evaluation was performed using the modified Lane-Sandhu Score. Osseous consolidation was assessed at six and 12 months postoperatively. Additionally, patient-reported outcomes and variations of surgical treatment were analysed. The prevalence of non-unions in our study was 30.56% for the ulna, 50% for the radius, and 19.44% for both bones. 85% of patients received bone grafts, with 48% receiving cortico-cancellous bone grafts (CCBG) and 52% receiving cancellous bone from the iliac crest or femur. In total, 67% of patients demonstrated consolidation of the non-union within six months of surgical treatment, increasing to 89% after one year. Among patients who received CCBG, 86% showed consolidation at six months, rising to 100% at one year. Additionally, patient-reported outcome measures (PROMs), including VAS and SF-12 scores, showed overall improvement. A sufficiently long follow-up period is crucial for patients with forearm non-unions. To ensure consolidation, a follow-up period of at least six months to one year is recommended. Furthermore, the use of cortico-cancellous bone grafting combined with locking compression plates (LCP) appears to be an effective technique, providing stability during the healing process and achieving satisfactory bone healing one year postoperatively.

## Introduction

Non-unions of the forearm occur in 2–5.3% of all cases, with their prevalence varying according to the presence of multiple risk factors [1–6]. These risk factors can be categorized as either patient-related factors, such as smoking, age, obesity, diabetes, and vascular disorders, or patient-independent factors, including open fractures, local infections, soft tissue conditions and defect size [7]. The failure of fracture healing in the forearm region can result in substantial limitations to the patient’s physical function, thus requiring a comprehensive treatment approach [8–11].

One of these treatment options is the Diamond concept, first introduced by Giannoudis et al. in 2007, first outlined four meanwhile outlines five key factors that define and significantly influence the complex interaction of bone healing [12]. These factors comprise osteogenesis, osteoinduction, osteoconduction, mechanical stability and vascularity [13]. Another frequently employed technique in the management of non-unions is the induced membrane technique. Its applications extend beyond the treatment of infections to encompass the enhancement of vascularization. Therefore, the induced membrane technique is considered to be the preferred treatment for primarily atrophic, infected and large non-unions [14].

The complex anatomies of the radius and ulna lead to variations in treatment, as these bones form a functional unit with the wrist and elbow joints [6, 9, 15]. Consequently, it is crucial to restore function promptly after treatment. To achieve this, the use of corticocancellous bone grafts is often preferred, as they provide superior mechanical stability following surgical intervention.

While the existing research on this topic remains limited, several smaller studies have begun to offer valuable insights into the treatment of non-unions in the forearm. The published literature not only supports the use of the Masquelet technique but also highlights the efficacy of locking plates and bone grafts, regardless of whether the bone is vascularized [16–22].

Nevertheless, non-unions of the forearm are relatively rare in trauma surgery, resulting in a significant deficit in research, resulting in a lack of robust evidence to support effective treatment methods as well as patient reported outcomes.

The aim of this study was to assess the osseous consolidation of non-unions of the forearm after 6 months and one year, employing radiological scores and patient-reported outcomes as evaluation criteria.

## Materials and methods

### Patients

In this study, non-unions were defined according to the European Society of Tissue Regeneration in Orthopedics and Traumatology (ESTROT) as a fracture which cannot heal without further intervention [13]. The non-unions included were partly hypertrophic, but some were also initially avascular or infectious.

Between 2010 and 2021, a total of 42 patients with non-unions of the ulna and/or radius were treated in our department. Inclusion criteria were: age of minimum 18 years, written informed consent, the presence of a non-union in the forearm (either in the radius, ulna, or both), and surgical treatment of the non-union. A minimum follow-up period of six months was required.

Exclusion criteria included loss to follow-up (LTFU) before the six-month follow-up appointment and being underage at the time of treatment.

This study was conducted in accordance with the current Declaration of Helsinki and was approved by the local Ethics Committee (S-262/2017). All patients provided written informed consent and agreed to the study protocol.

## Surgical technique

All patients were treated according to established protocols previously described by our department [13]. Treatment was performed using either a one-step technique or a two-step procedure known as induced membrane technique. In both cases, the presence of sufficient vascularity, as described in the table located in the appendix, was determined by the observation of adequate blood perfusion at the bone margins following bone debridement.

The one-step technique was employed in cases where the defect was minor or in the absence of infection in the preoperative performed contrast enhanced ultrasound (CEUS). The two-step procedure was utilized when infection was present, when the defect was avascular or large in size in order to improve vascularisation.

Bone grafts were utilized in the form of cancellous bone grafts or cortico-cancellous bone grafts harvested from the iliac crest in cases of larger defects (> 1,25 cm) or if further stabilisation was needed. Additionally, all patients were treated with bone substitutes such as tricalcium phosphate (TCP) (Vitoss^®^, Mahwah, NJ, USA), bioactive glass (BaG) (Bioglass^®^, Bonalive Biomaterials Ltd., Turku, Finland) or a combination of both (Vitoss-BA^®^, Mahwah, NJ, USA). In some cases, Cerabone and allogenic bone substitutes were also used, along with growth factors such as rhBMP-2 and rhBMP-7. All of the aforementioned bone substitutes and growth factors have been utilised in accordance with the current research consensus. In terms of osteosynthesis, locking compression plates (LCP) were used in all cases to ensure stability.

## Postoperative assessment, radiographic evaluation

Follow-up appointments were conducted at six weeks, twelve weeks, six months, and twelve months after surgery. At each visit, radiographic evaluations and physical examinations were performed by an experienced senior orthopedic surgeon.

The modified Lane-Sandhu Score (LSS), as shown in Table 1, was used to assess osseous consolidation. A score of three or higher was considered stable, while a score of four indicated complete osseous consolidation [23, 24].

**Table 1:** Modified lane-sandhu score (LSS) for radiological evaluation

Lane-sandhu-score	Radiological findings
0 1 2 3 4	No callus Minimal callus Callus evident, but healing incomplete Callus evident with stability expected Complete healing with bone remodeling

A standardized patient questionnaire was used to collect data for additional scores, including the visual analog scale (VAS) [25] and the short-form 12 (SF-12) [26].

The physical examination aimed to assess the range of motion (ROM) in the wrist and elbow joints. For wrist mobility, a moderate limitation was defined as a restriction of 20° or more in extension/flexion, while a restriction of less than 20° was considered a mild limitation. For pronation and supination, a mild limitation was defined as a restriction of less than 30°, while a moderate limitation was noted for restrictions greater than 30°. For extension and flexion in the elbow joint, a restriction of up to 30° was classified as mild, and a restriction exceeding 30° was classified as moderate.

### Data analysis

Statistical analysis was performed using IBM SPSS Statistics 29.0 and Stata statistical software (version 18.0, StataCorp, United States). Data were described using means and standard deviations or medians and interquartile ranges (IQR), as appropriate. Absolute numbers and percentages were used to illustrate distributions. Given the relatively small sample size, it was assumed that the data were not normally distributed. Therefore, the Wilcoxon signed-rank test was employed to compare the two groups. Categorical data were analyzed using the Fisher exact test. A p-value of < 0.05 was considered statistically significant.

## Results

### Patient characteristics

**Fig. 1:**
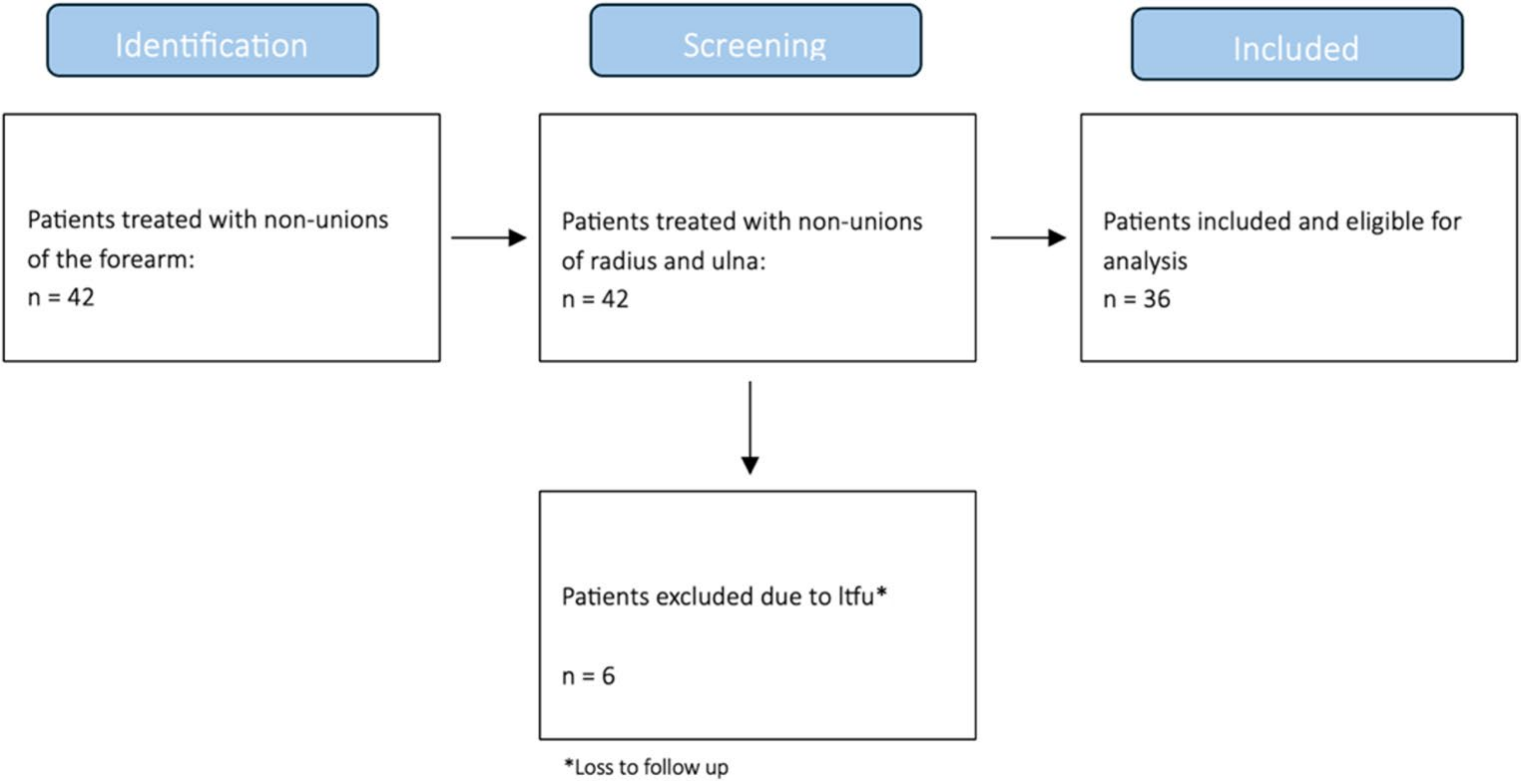
Screening Process

Of the 42 patients who met the inclusion criteria, six were excluded from the study due to loss to follow-up (*n* = 6) (Fig. 1).

The median age of the patients was 45.5 years (IQR: 29.5–59.5 years). The study cohort comprised 22 male and 14 female patients. Patient-related general risk factors are summarized in Table 2.

**Table 2:** Patient characteristics (ASA = American society of Anesthesiologists, IQR = interquartile range)

Factor	Level	Value
Age, median (IQR)		45.5 (29.5, 59.5)
Sex	Female	14 (39%)
	Male	22 (61%)
ASA Classification	1	13 (36%)
	2	20 (56%)
	3	3 (8%)
Smoking	No	24 (67%)
	Yes	12 (33%)
Diabetes	No	31 (86%)
	Yes	5 (14%)
BMI, median (IQR)		26 (23.75, 28.25)
Alc. abuse	No	36 (100%)
Infection	No Yes	30 (83%) 6 (17%)

Of the remaining 36 patients, 18 (50%) had non-unions of the radius, 11 (31%) had non-unions of the ulna, and 7 (19%) had non-unions involving both bones.

With regard to the initial cause, 34 patients sustained a fracture. Of the remaining two patients (out of a total of 36) one had congenital and one an iatrogenic non-union. Regarding fracture type, 26 patients (77%) had sustained an initial closed fracture, while 8 patients (23%) had an open fracture. Based on the Tscherne and Oestern classification system, 4 of these 8 patients (50%) had a grade 3 open fracture, 2 (25%) had a grade 2 open fracture, and 2 (25%) had a grade 1 open fracture.

The mean number of previous surgical interventions prior to non-union treatment at our department was 1.78 ± 0.96. Of all patients, 25 (69%) underwent a single-stage procedure, while the remaining patients (31%) were treated with a two-stage procedure.

In total, a median defect size of 0,68 cm (IQR: 0,45 − 1,39 cm) was observed. Overall, the median size of defects prior to Masquelet Step II in patients who underwent a two- or multistep procedure was found to be 1.59 cm (IQR: 1.31–2.30 cm).

## Surgical procedure

Seventeen patients (47%) received bone substitutes (Vitoss^®^, Bioglass^®^, Cerabone^®^, Vitoss-BA^®^, Vitoss BA2X^®^). Of these, 10 patients (59%) were additionally provided with a bone graft. Among the 29 patients treated with bone grafts, 15 received a cortico-cancellous bone graft (ccbg) (52%), while the remaining 14 patients were given a cancellous bone graft (cbg) (48%).

A total of 32 patients (89%) underwent plate osteosynthesis using a locking compression plate (LCP), while 4 patients (11%) retained their previous plate osteosynthesis without undergoing implant changes during treatment. Additionally, 9 patients (25%) received treatment with bone morphogenic protein (BMP).

In total, eight patients (22%) required at least one soft tissue revision surgery, and four patients (11%) underwent revision surgeries involving the bone. The specific reasons for the revisions are summarized in Table 3.

**Table 3:** Surgical procedure (cbg = cancellous bone graft, ccbg = cortico-cancellous bone graft, LCP = locking compression plate, TEN = titanium elastic nail)

Factor	Level	Value
N		36
Surgical procedure	Single step	26(72%)
	Multi-step	10 (28%)
Bone graft	None	7 (19%)
	cbg	14 (39%)
	ccbg	15(42%)
Bone substitute	Vitoss	9(25%)
	Vitoss BA2X	2 (6%)
	Vitoss BA	2(6%)
	Bioactive glass	2(6%)
	allogenic	2 (6%)
	none	19 (53%)
Prior fixation method	LCP	34 (94%)
	TEN	1 (3%)
	None	1 (3%)
Change of implant	Yes	32 (32%)
	No	4 (11%)
BMP	Yes	9 (25%)
	No	27 (75%)

As illustrated in Appendix A, the individual patients are shown alongside the respective columns of the Diamond Concept, which have been applied to them.

## Follow-up and radiographic outcomes

**Fig. 2:**
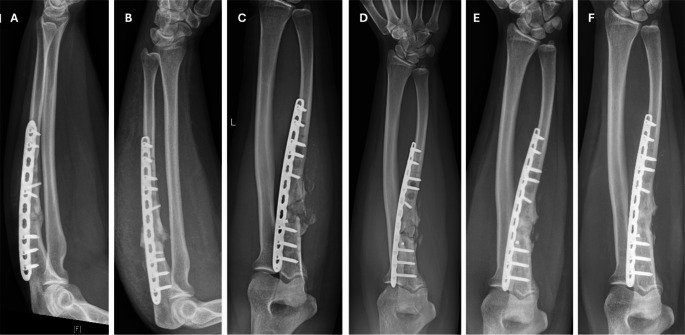
An example of an ulna non-union case is illustrated, accompanied by X-rays taken at various follow-up intervals

An example of a proximal ulna non-union with radiological follow-up at one year is presented in Fig. 2.

(A) Preoperative X-ray showing an infected non-union of the proximal ulna in a 26-year-old female patient, with a defect size of approximately 12 mm.
(B) Postoperative X-rays following the first surgical treatment, which involved the induced membrane technique, osteosynthesis with a 3.5 mm locking compression plate (LCP), radical debridement, and sequestrectomy of the sclerotic bone. A G-/V-PMMA spacer was locally applied.
(C) X-rays after the second surgical stage: the spacer was removed, and the osseous defect was augmented using autologous bi-cortico-cancellous bone graft harvested from the iliac crest, combined with bioactive glass (Bioglass^®^, Bonalive Biomaterials Ltd.).
(D) X-rays taken six weeks post-surgery showing initial callus formation with a Lane-Sandhu Score (LSS) of 1.
(E) X-rays at six months post-surgery demonstrating progressive callus formation and healing with expected stability (LSS 3).
(F) X-rays one year postoperatively indicating full osseous consolidation with an LSS of 4.**”**

Regarding radiological outcomes, six months post-op 24 patients (67%) reached consolidation, implying a LSS of three (47%, *n* = 17) or four (19%, *n* = 7).

One year after non-union treatment 89% of non-unions were considered consolidated. 25 patients reached a LSS of four (69%), seven a LSS of three (19%).

LSS distribution is displayed in Table 4.

**Table 4:** Distribution of the Lane-Sandhu score after 6 and 12 months (LSS = Lane-Sandhu Score)

Factor	Level	Value
LSS (6 months)	1	3 (8%)
	2	9 (25%)
	3	17 (47%)
	4	7 (19%)
LSS (12 months)	2	4 (11%)
	3	7 (19%)
	4	25 (69%)

15 Patients (42%) treated with ccbg had a mean consolidation time of 6.0 months (IQR: 4.5–6.0.5.0). 14 patients (39%) treated with cbg had a mean consolidation time of 6.0 months (IQR: 6.0–6.0). Three patients (20%) treated with cbg failed to achieve an LSS of 3 or 4 and therefore could not be included in this analysis. With a mean time of 12 months (IQR: 4.5–12.0), the seven patients (19%) without bone graft had the longest time until consolidation and one of those patients (14%) did not reach consolidation.

After six months, thirteen out of 36 patients (36%) were allowed to bear full load. After 12 months 19 out of 25 Patients (76%) were allowed to fully use the limb.

Six patients presented with an infected non-union (17%). Six months after treatment, three of those (50%) showed delayed consolidation (LSS < 3). 12 months postoperatively only one patient remained without consolidation (17%). Three of the six patients with infected non-union (50%) were treated via Masquelet-technique with a mean time to consolidation of three months, one failing to reach consolidation. The remaining three patients were treated in a one-step procedure, resulting in a mean consolidation time of ten months.

Among all patients four did not achieve consolidation. Out of these, one (25%) was affected by diabetes mellitus, infection, and obesity class one. None of the four patients were found to have a history of alcohol or substance abuse. Regarding smoking status, 12 out of 36 patients (33%) were active smokers, 24 were non-smokers (67%). Two of the 12 patients with an active smoking status did not reach consolidation (17%).

Obesity class 3 did not show impact on consolidation in this study.

Table 5 shows the consolidation after six months in relation to the risk factors.

**Table 5:** Risk factors and consolidation

Risk factor	No consolidation (*n* = 12)	Consolidation (*n* = 24)	*P*-value
infection			
Yes	3 (25%)	3 (12%)	0.38
No	9 (75%)	21 (88%)	
Smoking			
Yes	6 (50%)	6 (25%)	0.16
No	6 (50%)	18 (75%)	
Diabetes			
Yes	3 (25%)	2 (8%)	0.31
No	9 (75%)	22 (92%)	
Obesity per magna			
Yes	0 (0%)	1 (4%)	1.00
No	12 (100%)	23 (96%)	
Alcohol Abuse			
No	12 (100%)	24 (100%)	1.00

### Scores

The median of the mSF12 six months postoperative was 54.6 (IQR: 34.8–55.9) in 15 patients and 12 postoperative 54.4 (IQR: 43.4–57.6) in 10 patients. The pSF12 had a median of 48 (IQR: 42.35–54.7) six months postoperative and a median of 52.55 (IQR: 42–55.3) 12 months postoperative with the same number of patients as in the mSF12.

Six months following surgery, 50% of patients reported no pain during full use of the limb, 22% experienced mild pain, 11% reported moderate pain, 11% suffered from severe pain, and 6% endured extreme pain according to the visual analogue scale (VAS). 12 months postoperative 58% of patients had no pain during full use of the limb, 25% of patients had mild pain, 8% of patients had moderate pain and 8% of patients suffered from extreme pain. All scores can be seen in Table 6. Regarding the ability of full usage of the limb, the results are shown in Table 7.

**Table 6:** Scores (SF12 = Short form 12, mSF12 = mental score, pSF12 = physical score, VAS = visual analog scale for pain)

	Level	Value
6 months mSF12, median (IQR)		54.6 (34.8, 55.9)
6 months pSF12, median (IQR)		47 (42, 55.9)
6 months VAS		
0–1	9 (50%)	
1–3	4 (22%)	
3–5	2 (11%)	
5–7	2 (11%)	
7–9	1 (6%)	
12 months mSF12, median (IQR)		54.4 (43.4, 57.6)
12 months pSF12, median (IQR)		52.55 (45, 55.3)
12 months VAS		
0–1	7(58%)	
1–3	3 (25%)	
3–5	1 (8%)	
7–9	1 (8%)

**Table 7:** Distribution of full use of limb among the patients

	Level	Value
6 months full use of limb	Yes	13 (36%)
	No	23 (64%)
12 months full use of limb	Yes	19 (76%)
	No	6 (24%)

### Range of motion

Regarding pronation and supination of the wrist after twelve months, one patient (4%) had moderate limitations, and two patients (8%) had severe restrictions. Only one patient (4%) remained with a significant limitation in extension and flexion of the wrist twelve months after surgery. After twelve months, only three patients showed a restriction of the range of motion in the Ex/Flex of their elbow. Table 8 provides an additional illustration of the range of motion after six and twelve months.

**Table 8:** Range of motion (E/F = Extension/Flexion, P/S = Pronation/Supination)

ROM	Elbow E/F	Wrist *P*/S	Wrist E/F
6 months			
None	18 (60%)	18 (58%)	26 (84%)
Low	13 (40%)	6 (19%)	4 (13%)
Moderate	0 (0%)	3 (10%)	0 (0%)
High	0 (0%)	4 (13%)	1 (3%)
12 months			
None	21 (88%)	17 (71%)	24 (96%)
Low	3 (12%)	4 (17%)	0 (0%)
Moderate	0 (0%)	1 (4%)	0 (0%)
High	0 (0%)	2 (8%)	1 (4%)

## Discussion

The objective of this study was to evaluate the osseus consolidation after 6 months and one year as well as the clinical outcome of patients with non-unions of the ulna and radius who underwent surgical treatment.

In our study an osseous consolidation rate of 67% at six months and 89% at 12 months was observed. Regarding the consolidation after six months, Regan et al. [27] published a retrospective study, involving 23 Patients with non-unions of the forearm. They evaluated the functionality of LCP and bone grafts as treatment option. After six months 78% reached consolidation and 100% after 12 months. 9% were treated with a second revision surgery after initial treatment in order to achieve consolidation. The average consolidation time was 6.3 months. Compared to the osseus consolidation rates in our study, Regan et al. found higher consolidation rates. This might be attributed to the fact that in their study, Regan et al. had only one patient with proven infection was included while six patients of our cohort had a positive intraoperative culture. Infection is a known risk factor for non-union [7, 17].

Regarding the consolidation after 12 months, Dhar et al. [18] published a prospective study involving 12 patients, presenting with an infected non-unions of the forearm. In this study, all patients were treated with the Masquelet technique, resulting in an osseus consolidation rate of 100% within 12 months. The average consolidation time reported was 7.8 months. Regarding the consolidation after 12 months in our study, 89% reached consolidation. In comparison to our consolidation rate of 89% after 12 months, the study by Dhar et al. demonstrates a higher consolidation rate of 100%. This discrepancy might be attributed to the fact that all patients in the aforementioned study were treated using the Masquelet technique [18]. Three patients in our group were treated with a single-stage procedure despite subsequent evidence of an infectious non-union, which might also explain prolonged or absent healing.

Regarding bone grafts, three patient cohorts were compared in this study: one treated with ccbg, another with cbg, and a third without any bone grafts. The cohort receiving ccbg achieved a consolidation time with a median of 6.0 months, with all patients in this group reaching full consolidation. In contrast, the cbg group had a consolidation time with a median of 6.0 months, with 80% consolidation after one year. A significant factor contributing to this rapid consolidation may be the additional stability provided by using a ccbg [28]. The group without any bone grafts had an extended consolidation time with a median of 12 months and included one patient who did not achieve consolidation within one year. This finding can be substantiated by the Diamond concept, which advocates the utilization of stem cells or bone grafts [12, 29].

Furthermore, the utilization of LCP is identified as a reliable element due to its capacity to offer substantial stabilization and bridging at the non-union site in this study, thus facilitating the healing process [20, 30, 31]. This is consistent with the findings of our study.

In general, some risk factors for delayed consolidation applied to several patients in this study, including smoking, obesity per magna, diabetes and intraoperative evidence of bacteria. Although those risk factors are proven to show high correlation with delayed bone healing [17, 32], the majority of patients affected in this study did not show any disadvantage in consolidation after the non-union treatment in our department. This excludes the cohort of patients affected by smoking. Out of 12 patients who were smokers, six patients did show delayed healing and remained without consolidation at 6 months postoperative. At 12 months postoperative two patients remained without consolidation, indicating that smoking might have affected the healing process. Due to the small patient collective those results could not be proved with statistical significance and only provide an estimation.

Regan et al. [27] evaluated in their study not only the osseus consolidation but also physical and functional outcomes of their non-union treatment, using the VAS and ROM. They showed that patients reached a significant reduction on the VAS in the course of therapy, which resulted in improved function. The study highlights that improvement in function is likely related to optimization of forearm length, alignment and rotation after surgery. These improvements contribute to better range of motion and stronger grip strength. These results are consistent with those of our study, in which an association between the improvement of PROMs and patient functionality could be observed during therapy. Generally, it can be stated that the evaluation of these patient-reported scores serves as a valuable complement for assessing the success of therapy.

The present study was limited by several factors. Firstly, it is a retrospective study and therefore causal dependencies cannot be demonstrated with complete certainty. Moreover, the patient cohort is limited due to the rarity of this specific location of non-unions. This limits the statistical analysis as well as the generalizability of the results, particularly in the context of sub-group analysis (for example regarding the use of bone grafts). Furthermore, conducting a precise analysis of bone substitutes and growth factors was not feasible due to the substantial number of these substances used. This phenomenon can be attributed the prolonged inclusion period and the evolution of the strategies over time according to the research conducted at the time. Furthermore, as some patients did not complete the full questionnaire, data regarding the SF-12 is limited.

## Conclusion

In conclusion, a satisfactory fusion rate can be seen in the treatment of non-unions of the forearm using ccbg or cbg in combination with LCP. Furthermore, looking at our results a follow-up period of at least six months, with a preference for 12 months should be standard of care treating non-unions of the forearm. Overall improvements in mobility were found to correlate positively with quality-of-life enhancements as indicated by pSF-12 scores.

## Data Availability

The datasets used and/or analysed during the current study are available from the corresponding author on reasonable request.

## Appendix A: overview of the surgical procedure for each individual patient

**Table 9:** Overview of the surgical procedure for each individual patient

Patient	Surgery (1 = one-step, 2 = two-/multiple-step)	Mechanical stability	Osteogenic cells	Scaffolds	Growth factors	Vascularity
1	1	X	X	X	X	X
2	1	X				X
3	2	X	X	X		X
4	2	X				X
5	2	X	X	X		X
6	1	X	X	X		X
7	2	X				X
8	1	X				X
9	1	X	X			X
10	2	X	X	X		X
11	1	X	X	X	X	X
12	1	X				X
13	1	X	X			X
14	1	X	X			X
15	1	X	X			X
16	2	X	X			X
17	2	X	X	X		X
18	1	X	X		X	X
19	2	X	X		X	X
20	1	X	X			X
21	1	X	X	X		X
22	2	X	X	X		X
23	2	X	X		X	X
24	1	X				X
25	1	X	X			X
26	1	X			X	X
27	1	X	X			X
28	1	X	X		X	X
29	1	X	X			X
30	1	X	X	X		X
31	1	X	X	X		X
32	1	X	X	X		X
33	2	X	X	X		X
34	1	X	X		X	X
35	1	X	X	X		X
36	1	X	X		X	X

## References

[1] Wei SY, et al. Diaphyseal forearm fractures treated with and without bone graft. J Trauma. 1999;46(6):1045–8. 10372622 10.1097/00005373-199906000-00011

[2] Wright RR, Schmeling GJ, Schwab JP. The necessity of acute bone grafting in diaphyseal forearm fractures: a retrospective review. J Orthop Trauma. 1997;11(4):288–94. 9258828 10.1097/00005131-199705000-00012

[3] Chapman MW, Gordon JE, Zissimos AG. Compression-plate fixation of acute fractures of the diaphyses of the radius and ulna. J Bone Joint Surg Am. 1989;71(2):159–69. 2918001

[4] Anderson LD, et al. Compression-plate fixation in acute diaphyseal fractures of the radius and ulna. J Bone Joint Surg Am. 1975;57(3):287–97. 1091653

[5] Ross ER, et al. Retrospective analysis of plate fixation of diaphyseal fractures of the forearm bones. Injury. 1989;20(4):211–4. 2592095 10.1016/0020-1383(89)90114-9

[6] Hadden WA, Reschauer R, Seggl W. Results of AO plate fixation of forearm shaft fractures in adults. Injury. 1983;15(1):44–52. 6885147 10.1016/0020-1383(83)90162-6

[7] Santolini E, West R, Giannoudis PV. Risk factors for long bone fracture non-union: a stratification approach based on the level of the existing scientific evidence. Injury. 2015;46:S8–19. 26747924 10.1016/S0020-1383(15)30049-8

[8] Shabir AD, et al. Delayed operative management of fractures of the lateral condyle of the humerus in children. Malays Orthop J. 2015;9(1):18–22. 28435590 10.5704/MOJ.1503.010 PMC5349342

[9] Skahen JR 3, et al. The interosseous membrane of the forearm: anatomy and function. J Hand Surg Am. 1997;22(6):981–5. 9471064 10.1016/S0363-5023(97)80036-6

[10] Hollister AM, Gellman H, Waters RL. The relationship of the interosseous membrane to the axis of rotation of the forearm. Clin Orthop Relat Res. 1994;298:272–6. 8118987

[11] Tarr RR, Garfinkel AI, Sarmiento A. The effects of angular and rotational deformities of both bones of the forearm. An in vitro study. J Bone Joint Surg Am. 1984;66(1):65–70. 6690445

[12] Giannoudis PV, et al. The diamond concept–open questions. Injury. 2008;39(Suppl 2):S5–8. 18804574 10.1016/S0020-1383(08)70010-X

[13] Schmidmaier G, Moghaddam A. Long bone nonunion. Z Orthop Unfall. 2015;153(6):659–74. quiz 675-6. 26670151 10.1055/s-0035-1558259

[14] Raven TF, et al. Use of masquelet technique in treatment of septic and atrophic fracture nonunion. Injury. 2019;50(Suppl 3):40–54. 31378541 10.1016/j.injury.2019.06.018

[15] LaStayo PC, Lee MJ. The forearm complex: anatomy, biomechanics and clinical considerations. J Hand Ther. 2006;19(2):137–44. 16713861 10.1197/j.jht.2006.02.002

[16] Walker M, Sharareh B, Mitchell SA. Masquelet reconstruction for posttraumatic segmental bone defects in the forearm. J Hand Surg Am. 2019;44(4):e3421–8. 10.1016/j.jhsa.2018.07.003 30146386

[17] Jensen SS, et al. Risk factors for nonunion following surgically managed, traumatic, diaphyseal fractures: a systematic review and meta-analysis. EFORT Open Rev. 2022;7(7):516–25. 35900220 10.1530/EOR-21-0137 PMC9297052

[18] Dhar SA, Dar TA, Mir NA. Management of infected nonunion of the forearm by the masquelet technique. Strategies Trauma Limb Reconstr. 2019;14(1):1–5. 32559259 10.5005/jp-journals-10080-1411 PMC7001591

[19] Choi SW, et al. Treatment of forearm diaphyseal non-union: autologous Iliac corticocancellous bone graft and locking plate fixation. Orthop Traumatol Surg Res. 2021;107(8):102833. 33524631 10.1016/j.otsr.2021.102833

[20] Ring D et al. Locking compression plates for osteoporotic nonunions of the diaphyseal humerus. Clin Orthop Relat Res, 2004(425): pp. 50–4. 10.1097/01.blo.0000131484.27501.4b 15292787

[21] Kloen P, Buijze GA, Ring D. Management of forearm nonunions: current concepts. Strategies Trauma Limb Reconstr. 2012;7(1):1–11. 22113538 10.1007/s11751-011-0125-0 PMC3332319

[22] Soucacos PN, et al. Vascularised bone grafts for the management of non-union. Injury. 2006;37(Suppl 1):S41–50. 16581074 10.1016/j.injury.2006.02.040

[23] Lane JM, Sandhu HS. Current approaches to experimental bone grafting. Orthop Clin North Am. 1987;18(2):213–25. 3550572

[24] Schnetzke M, et al. Additional bone graft accelerates healing of clavicle non-unions and improves long-term results after 8.9 years: a retrospective study. J Orthop Surg Res. 2015;10:2. 25573541 10.1186/s13018-014-0143-y PMC4296679

[25] Aitken RC. Measurement of feelings using visual analogue scales. Proc R Soc Med. 1969;62(10):989–93. 4899510 10.1177/003591576906201005 PMC1810824

[26] Ware J, Kosinski M, Keller S. *SF-12: How to Score the SF-12 Physical and Mental Health Summary Scales.* 1998.

[27] Regan DK, et al. Functional outcomes of compression plating and bone grafting for operative treatment of nonunions about the forearm. J Hand Surg Am. 2018;43(6):564 e1-564 e9. 29224947 10.1016/j.jhsa.2017.10.039

[28] Giannoudis PV, Dinopoulos H, Tsiridis E. Bone substitutes: an update. Injury. 2005;36:S20–7. 16188545 10.1016/j.injury.2005.07.029

[29] Miska M, Schmidmaier G. [Diamond concept for treatment of nonunions and bone defects]. Unfallchirurg. 2020;123(9):679–86. 32761357 10.1007/s00113-020-00843-1

[30] Ring D, et al. Ununited diaphyseal forearm fractures with segmental defects: plate fixation and autogenous cancellous bone-grafting. J Bone Joint Surg Am. 2004;86(11):2440–5. 15523016

[31] Kumar MN, Ravindranath VP, Ravishankar M. Outcome of locking compression plates in humeral shaft nonunions. Indian J Orthop. 2013;47(2):150–5. 23682176 10.4103/0019-5413.108899 PMC3654464

[32] Bishop JA, et al. Assessment of compromised fracture healing. J Am Acad Orthop Surg. 2012;20(5):273–82. 22553099 10.5435/JAAOS-20-05-273

